# Anxiety and Depressive Disorders in Chronic Hemodialysis Patients

**DOI:** 10.1192/j.eurpsy.2026.11421

**Published:** 2026-07-31

**Authors:** H. Hsine, S. Kolsi, A. Bouaziz, W. Abbes, M. El Behi

**Affiliations:** 1 Psychiatry, La Metairie Clinic, Nyon, Switzerland; 2 Psychiatry, University Hospital of Gabès, Gabes, Tunisia

## Abstract

**Introduction:**

Chronic kidney disease and the need for hemodialysis profoundly impair patients’ quality of life and promote the onset of psychiatric disorders, particularly anxiety and depression. These anxiety-depressive disorders are frequent, often underdiagnosed, and worsen both morbidity-mortality and treatment adherence.

**Objectives:**

Our aim is to determine the prevalence of anxiety and depression in hemodialysis patients while identifying the associated factors.

**Methods:**

A descriptive and analytical cross-sectional study was conducted at the University Hospital of Gabès between February and May 2025. The study population included 33 chronic hemodialysis patients for more than 3 months. Anxiety and depression were screened using the HADS (Hospital Anxiety and Depression Scale). Sociodemographic, clinical, and associated factors were also analyzed.

**Results:**

The study included 73 chronic hemodialysis patients, with a mean age of 46.4 ± 14.4 years, ranging from 16 to 74 years. The population comprised 51.5% women, with a male-to-female sex ratio of 0.94. Psychological evaluation using the HADS revealed that 30.3% of participants had anxiety symptoms (HADS-A ≥ 8) and 24.2% had depressive symptoms (HADS-D ≥ 8). Depression was significantly correlated with a low socioeconomic level (p=0.012) and difficulty accessing the dialysis center (p=0.01). Moreover, low family support was associated with impaired physical quality of life, with a PCS-12 score below 50 (p=0.027).

**Conclusions:**

Anxiety and depressive disorders are frequent among hemodialysis patients. These conditions have a major social impact and require appropriate management. Systematic screening using the HADS scale and multidisciplinary care, including psychological and social support, are essential to improve prognosis and quality of life in dialysis patients.

**Disclosure of Interest:**

None Declared

